# Pulmonary Hemorrhage Secondary to Disseminated Strongyloidiasis in a Patient with Systemic Lupus Erythematosus

**DOI:** 10.1155/2015/310185

**Published:** 2015-05-26

**Authors:** Erika P. Plata-Menchaca, V. M. De la Puente-Diaz de Leon, Adriana G. Peña-Romero, Eduardo Rivero-Sigarroa

**Affiliations:** ^1^Department of Critical Care Medicine, Instituto Nacional de Ciencias Médicas y Nutrición Salvador Zubirán (INCMNSZ), 14000 Mexico City, DF, Mexico; ^2^Department of Dermatology, Instituto Nacional de Ciencias Médicas y Nutrición Salvador Zubirán (INCMNSZ), 14000 Mexico City, DF, Mexico

## Abstract

*Introduction*. Pulmonary hemorrhage secondary to disseminated strongyloidiasis is an unusual, well-recognized entity in immunocompromised patients with autoimmune disease, which is associated with the hyperinfection syndrome, sepsis, and a high mortality rate. *Case Presentation*. We present a case of a 44-year-old Mexican woman with systemic lupus erythematosus and acute bacterial meningitis who developed pulmonary hemorrhage with acute respiratory failure requiring mechanical ventilation, treated with broad spectrum systemic antibiotics and high dose methylprednisolone, who subsequently developed a characteristic purpuric skin eruption and septic shock and died two days later of refractory hypoxemia caused by massive pulmonary bleeding. The postmortem examination reports filariform larvae of *S. stercolaris* in lung, skin, and other organs. *Conclusion*. This case highlights the importance of considering disseminated strongyloidiasis in the differential diagnosis of diffuse alveolar hemorrhage in systemic lupus erythematosus, and screening for *S. stercolaris* infection before initiation of immunosuppressive therapy should be considered, especially in endemic areas. Disseminated strongyloidiasis has a high mortality rate, explained in part by absence of clinical suspicion.

## 1. Introduction

Disseminated strongyloidiasis (DS) is the systemic infection in immunocompromised hosts of* Strongyloides stercoralis*, a parasite that commonly causes limited gastrointestinal disease in immunocompetent hosts.* S. stercoralis* is a widespread, soil-transmitted intestinal nematode common in tropical and subtropical areas affecting the lower socioeconomic groups [1]. Risk factors associated with DS are those that affect cellular immunity, such as hematologic malignances, HTLV-1 infection, bone marrow transplantation, anti-TNF medication, HIV, and glucocorticoids [2]. The latter associated with impairment of the intestinal wall thickness, concomitant bacterial translocation, and provoking superinfection syndrome, which refers to the widespread dissemination of the parasites from the gut to diverse organs [3]. Approximately 30% of cases of DS have been reported in immunocompromised patients with autoimmune disease. The diagnosis is usually made in concentrated repeated stool examinations; serial testing is recommended to increase the sensitivity of the diagnostic test [4], although the serological testing by the ELISA method is the best option for this purpose [5].

## 2. Case Presentation

A 44-year-old woman, from Veracruz, Mexico, of low socioeconomic income, was admitted to the hospital with one-week history of progressive headache, phonophobia, high-grade fever, vomiting, and watery diarrhea. She had systemic lupus erythematosus (SLE) since 2003, with cutaneous, joint, and renal involvement. In the previous month, SLE-associated thrombotic thrombocytopenic purpura (TTP) was diagnosed and was treated with plasmapheresis, cyclophosphamide, and 1 mg/kg/day of prednisone.

**Figure 1:**
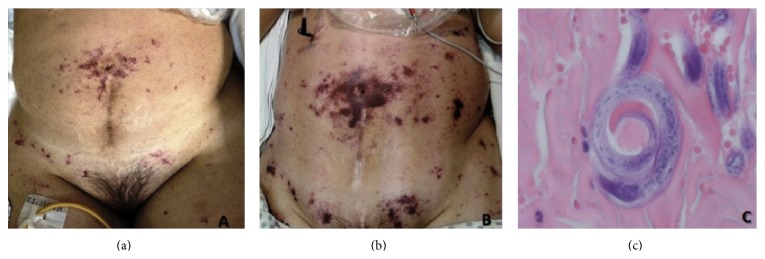
Purpuric dermatosis and skin biopsy. Punctiform periumbilical purpuric macules (a). 24 hours later, dermatosis worsened and was confluent with persistence of periumbilical involvement (*“Thumb print sign”*) (b). Filariform larvae were observed in the dermis (c).

**Figure 2:**
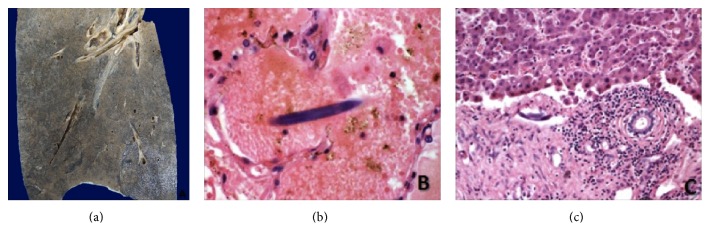
Postmortem examination. Macroscopic appearance of lung (a).* S. stercoralis* filariform larvae in lung (b) and liver (c).

At admission, she had systemic inflammatory response syndrome and nuchal rigidity; focal neurologic signs and clinical signs of lupus activity were absent. Laboratory evaluation showed Hb 11.2 gr/dL, MCV 103 fL, eosinophil count 150 cel/mm^3^, serum creatinine 0.8 mg/dL, haptoglobins 77 mg/dL (>30 mg/dL), ALT 59 UI/L (<34), AST 78 UI/L (<40), LDH 310 (<270 mg/dL), cobalamin levels 406 pg/mL (>150 pg/mL), folates 49.6 ng/mL (>4 ng/mL), C3 96.5 mg/dL (87–200 mg/dL), lactate 0.9 (<2.2 mmol/L), and mild proteinuria (310 mg/day). The initial chest X-ray and head-CT did not show any abnormalities. The lumbar puncture revealed pH 7.35, glucose 45 mg/dL (serum glucose 130 mg/dL), proteins 329 mg/dL, 42 white blood cells/mm^3^ (95% neutrophils), and 2 red blood cells/mm^3^.* Enterococcus gallinarum* was isolated in cerebrospinal fluid and intravenous ampicillin was administrated with improvement in the neurological symptoms. On day 3, she developed hemoptysis with bilateral patchy pulmonary infiltrates on chest X-ray, a drop in hemoglobin concentration, and acute respiratory failure, which needed mechanical ventilation and admission to the intensive care unit (ICU). We obtained cultures and started methylprednisolone boluses and broad spectrum antibiotics. The initial clinical diagnosis was diffuse alveolar hemorrhage caused by SLE activity without sufficient clinical criteria of nosocomial pneumonia. On day 4 she developed a purpuric skin rash and a biopsy was performed (Figure 1). The additional approach did not show any evidence of hemolysis, disseminated intravascular coagulation, nor TTP. One day later she had clinical deterioration with septic shock and progression of the radiographic infiltrates. On day 6 she died because of refractory hypoxemia secondary to massive pulmonary bleeding. Filariform larvae of* S. stercoralis* were observed in the skin biopsy and postmortem examination report was SLE and DS with lung, thyroid, liver, gut, and skin involvement (Figure 2).

## 3. Discussion

Common presenting symptoms of acute disseminated strongyloidiasis in immunocompromised hosts include fever, abdominal pain, diarrhea, neurologic involvement, skin lesions, myositis, acute respiratory failure, and sepsis. The pathognomonic rash of* Strongyloides* infection is serpiginous and urticarial “larva currens” [5, 6]. Rarely, in DE, a progressive petechial purpuric eruption over the abdominal and proximal thigh, the “thumbprint purpura” (Figure 1) [7], is observed in absence of eosinophilia [8].

Evidence of superimposed bacterial infections that complicate the clinical course of patients, such as* Pseudomonas aeruginosa* and* Acinetobacter* pneumonia and* Enterococcus faecalis* meningitis, has been described [8, 9] which often progress to septic shock and are the direct cause of death in 80% of cases.

Differential diagnosis of DS includes SLE flare, vasculitis, inflammatory bowel disease [10], other systemic infections, and drug reactions. Treatment of DS has been done with ivermectin and albendazole, showing cure rate for immunocompromised patients of approximately 60%–70% [11–13].

The most common cause of diffuse alveolar hemorrhage in SLE is pulmonary capillaritis, often associated with bacterial pneumonia. The mortality rate has been declined since the rapid recognition and beginning of aggressive treatment with high dose methylprednisolone and cyclophosphamide. Because immunosuppressive therapy may provoke superinfection syndrome, screening for* S. stercoralis* infection before initiation of immunosuppressive therapy should be considered, especially in endemic areas [5–9].

## 4. Conclusion

Pulmonary hemorrhage secondary to DS is an unusual, well-recognized entity in immunocompromised patients that is associated with a high mortality rate, explained by disease severity and absence of clinical suspicion. It should be considered in the differential diagnosis of pulmonary hemorrhage in a patient with SLE before initiating steroids and especially when the clinical status worsens rapidly with steroids.
